# Longitudinal Changes in Inflammation-Related Methylation Risk Score for C-Reactive Protein Among People with HIV

**DOI:** 10.3390/epigenomes10030054

**Published:** 2026-08-12

**Authors:** Yijie Huang, Junyu Chen, Alexandra Young, Qin Hui, Johnathan A. Edwards, Alec Powers, Matthew R. Dudgeon, Selvan Pillay, Jaysingh Brijkumar, Mahomed Y. S. Moosa, Marta Gwinn, Viola Vaccarino, Vincent C. Marconi, Yan V. Sun

**Affiliations:** 1 Department of Epidemiology, Rollins School of Public Health, Emory University, Atlanta, GA 30322, USA; yijie.huang@emory.edu (Y.H.); junyu.chen@emory.edu (J.C.); alexandra.young@emory.edu (A.Y.); qhui@emory.edu (Q.H.); alec.powers@northwestern.edu (A.P.); marta.gwinn@emory.edu (M.G.); lvaccar@emory.edu (V.V.); 2 Department of Biostatistics and Bioinformatics, Rollins School of Public Health, Emory University, Atlanta, GA 30322, USA; alex.edwards@emory.edu; 3 Division of Epidemiology and Biostatistics, Faculty of Medicine and Health Sciences, Stellenbosch University, Cape Town 8000, South Africa; 4 Impact Institute, Feinberg School of Medicine, Northwestern University, Chicago, IL 60611, USA; 5 Department of Medicine, School of Medicine, Emory University, Atlanta, GA 30322, USA; 6 Department of Infectious Diseases, Nelson R Mandela School of Medicine, University of KwaZulu-Natal, Durban 4001, South Africa; selvan@adrenergy.com (S.P.); jbrijkumar@gmail.com (J.B.); moosay@ukzn.ac.za (M.Y.S.M.); 7 Adrenergy Research Innovations, Durban 4001, South Africa; 8 Veterans Affairs Atlanta Healthcare System, Decatur, GA 30033, USA; 9 Hubert Department of Global Health, Rollins School of Public Health, Emory University, Atlanta, GA 30322, USA

**Keywords:** inflammation, HIV, DNA methylation, methylation risk score, longitudinal study

## Abstract

**Background/Objectives**: Human immunodeficiency virus (HIV) infection is characterized by chronic systemic inflammation. Antiretroviral therapy (ART) can reduce persistent inflammation, as measured by C-reactive protein (CRP). The CRP methylation risk score (MRS_CRP_) acts as proxy for plasma CRP, but longitudinal changes in this score for people with HIV (PWH) before and after initiating ART have not been described. **Methods**: We evaluated a previously published MRS_CRP_ in the Emory Twin Study (N = 352), testing its correlation with plasma CRP and CRP-responsive biomarkers, as well as associations with cardiometabolic traits using generalized estimating equations (GEEs). We then applied MRS_CRP_ in the HIV AIDS Drug Resistance Surveillance Study (ADReSS) cohort (N = 440) to assess longitudinal changes before and after ART, using paired *t*-tests and linear mixed-effects models. **Results**: MRS_CRP_ showed moderate correlation with CRP (r = 0.38) and other inflammatory biomarkers (r = 0.25–0.34). MRS_CRP_ was associated with hypertension, type 2 diabetes, coronary artery disease, and obesity, and remained independently associated with obesity (odds ratio = 1.49) after adjusting for measured CRP. Z-score-standardized MRS_CRP_ decreased by 0.254 standard deviation units between baseline and follow-up among PWH receiving ART, confirmed by linear mixed-effects models. **Conclusions**: MRS_CRP_ showed associations with chronic inflammation and cardiometabolic diseases. The observed decline in MRS_CRP_ following ART initiation may reflect changes in inflammatory status among PWH. These findings highlight the potential of MRS_CRP_ as a marker of inflammation-related changes and comorbidity risk in PWH.

## 1. Introduction

More than 1.2 million people in the United States are living with human immunodeficiency virus (HIV) infection [1]. Untreated HIV infection can lead to significant CD4+ T-cell depletion and dysfunction, ultimately leading to acquired immune deficiency syndrome (AIDS) [2]. Over the past several decades, antiretroviral therapy (ART) has substantially reduced the global burden of AIDS by improving immune function and suppressing HIV viral replication [3,4]. Although ART can delay progression to AIDS, people with HIV (PWH) frequently experience comorbidities during long-term treatment [5].

Inflammation is an independent predictor of HIV progression and a risk factor for HIV-related comorbidities [6,7]. Chronic low-grade inflammation induced by persistent HIV infection contributes to inflammatory cytokine elevation, which increases the risk of cardiometabolic diseases [8,9,10], including acute myocardial infarction [9] and chronic obstructive pulmonary disease (COPD) [11].

C-reactive protein (CRP) is a biomarker that provides information about an individual’s chronic inflammatory status, distinct from traditional markers of HIV infection progression, such as CD4 count and viral load [12]. CRP levels can be reduced during ART [13]; therefore, monitoring CRP levels could enhance the prediction of HIV-related cardiometabolic diseases and mortality [9,14,15]. However, the use of CRP as a marker of ART-related inflammatory changes is complicated by its sensitivity to acute conditions such as infection, trauma, liver dysfunction, and psychological stress [16,17,18,19,20]. This variability could complicate the assessment of inflammatory recovery from ART in PWH, reducing the utility of CRP as a biomarker for risk of chronic diseases attributable to inflammation.

DNA methylation is an epigenetic modification that regulates gene expression without changing the underlying DNA sequence, primarily occurring at cytosine-phosphate-guanine (CpG) sites. In a cell-based epigenomic study, DNA methylation changes induced by the HIV trans-activator of transcription (Tat) protein were associated with inflammatory regulation in PWH [21]. An epigenome-wide association study (EWAS) that examined DNA methylation patterns at CpG sites across the genome revealed unique inflammation-related signatures among PWH [22]. Recently, several large, multi-ancestry EWAS have discovered CpG sites associated with plasma CRP levels [23,24,25,26]. These results are the basis for CRP methylation risk scores (MRS_CRP_) that have been built using advanced statistical and machine-learning models. In several cross-sectional studies, MRS_CRP_ demonstrated stronger associations than plasma CRP with cardiometabolic diseases, sleep outcomes, and COPD [23,24,27]. However, MRS_CRP_ has not been investigated for its potential to monitor longitudinal, chronic inflammatory changes.

MRS_CRP_ has better temporal stability than plasma CRP, as demonstrated by its higher intraclass correlation coefficient in a longitudinal setting [28]. By capturing an epigenetic signature of CRP, MRS_CRP_ may provide a more robust measure of cumulative inflammation in individuals with chronic infections like HIV. In this study, we aimed to evaluate the use of MRS_CRP_ in a longitudinal cohort of PWH. We utilized three previously published MRS_CRP_ derived from large multi-ancestry cohorts to maximize generalizability and assess their potential utility in capturing inflammation-related changes following ART. We hypothesized that MRS_CRP_ could be a potential indicator of chronic, low-grade inflammatory changes in PWH before and after ART.

## 2. Results

### 2.1. MRS_CRP_ Evaluation in the Emory Twin Study

**Figure 1:**
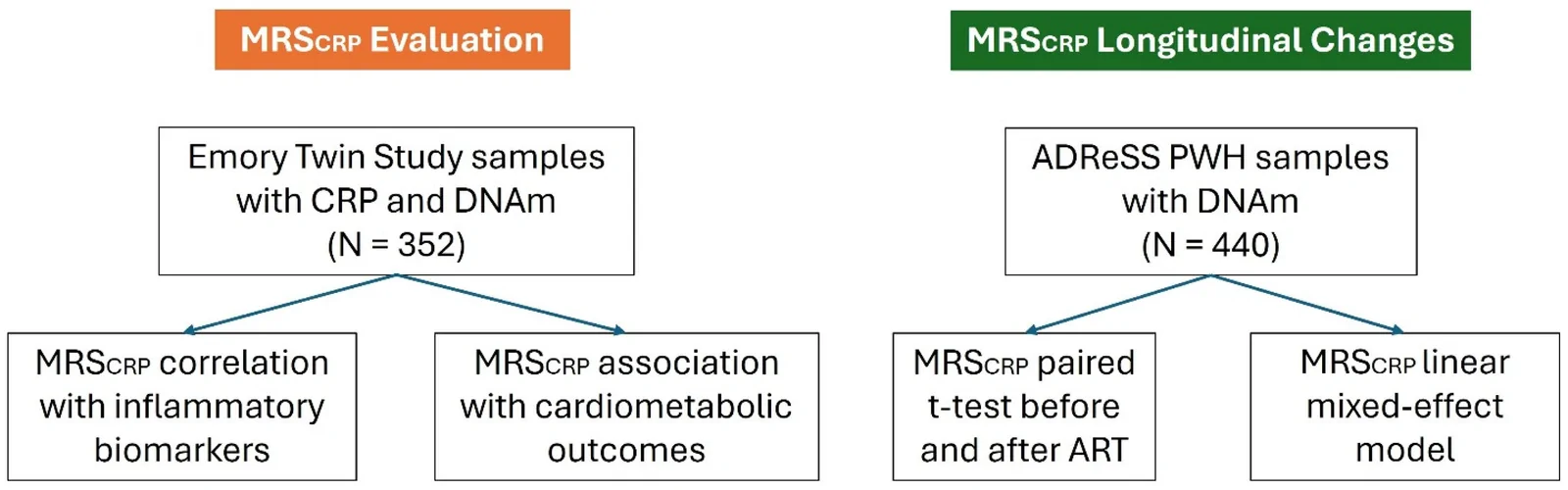
Overview of study design and workflow.

As outlined in the study design (Figure 1), we first evaluated three previously published MRS_CRP_ derived from large multi-ancestry EWAS in the Emory Twin Study to identify the best-performing score. The MRS_CRP_ included MRS_CRP_ developed from an EWAS by *Wielscher et al.* (MRS_CRP_W_) [23] and MRS_CRP_ developed from *Hillary et al.*’s EWAS using (1) a Bayesian penalized-regression-based model (MRS_CRP_B_) and (2) an elastic net regression-based model (MRS_CRP_E_) [24]. Demographic and clinical information for the Emory Twin Study participants is presented in Table 1. A total of 352 male participants were included in the evaluation of MRS_CRP_, with an average age of 55.7 years. Most participants were non-Hispanic white. Approximately 27% of the study population were current smokers. The mean body mass index (BMI) was 29.8 kg/m^2^. The median and interquartile range (IQR) of plasma high-sensitivity CRP and triglyceride levels were 1.5 mg/L (2.6 mg/L) and 161.0 mg/dL (109 mg/dL). Clinical traits among participants included obesity (45%), type 2 diabetes (12.8%), and prevalent coronary artery disease (CAD) (11.4%). 

**Figure 2:**
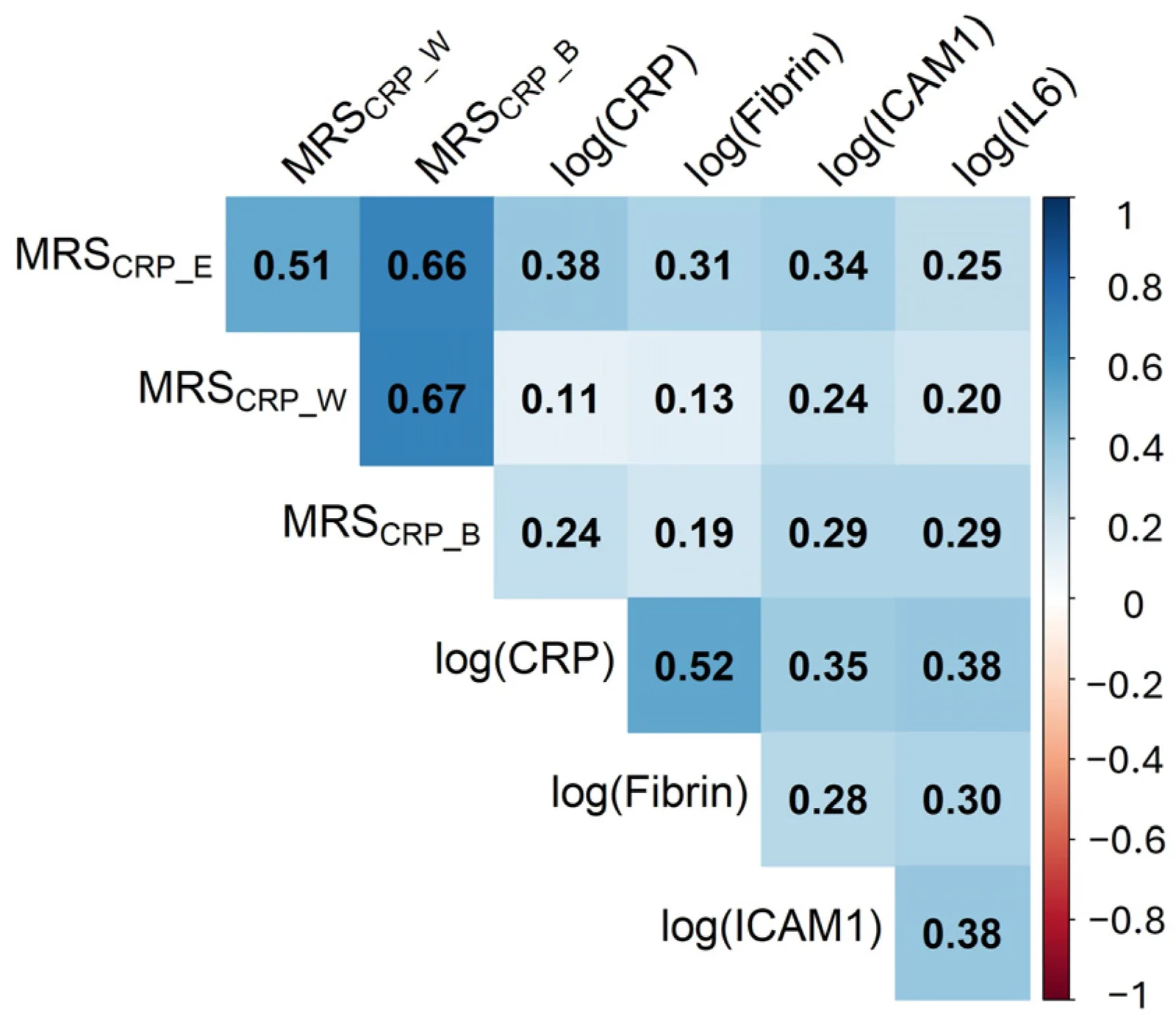
Pearson correlation matrix between calculated MRS_CRP_ and plasma level of other inflammatory biomarkers in the Emory Twin Study, while accounting for twin pairs. Due to skewed data for these biomarkers, each was transformed (natural log transformation) to obtain a normal distribution. The values in each cell represent Pearson correlation coefficients and are shown in gradient colors.

Three MRS_CRP_ (MRS_CRP_W_, MRS_CRP_B_, and MRS_CRP_E_) with different weights were calculated for participants in the Emory Twin Study. Among the three MRS_CRP_, MRS_CRP_E_ showed the strongest correlation with natural log-transformed plasma CRP, with a Pearson correlation coefficient of 0.38. In addition, MRS_CRP_E_ correlated most strongly with pro-inflammatory markers fibrin and Intercellular Adhesion Molecule 1 (ICAM-1) (Pearson correlation coefficients 0.31 and 0.34, respectively). Interleukin-6 (IL-6) was slightly less strongly correlated with MRS_CRP_E_ compared with MRS_CRP_B_. All MRS_CRP_ showed weaker correlations with other inflammatory markers compared with plasma CRP. The full results are summarized in Figure 2.

**Figure 3:**
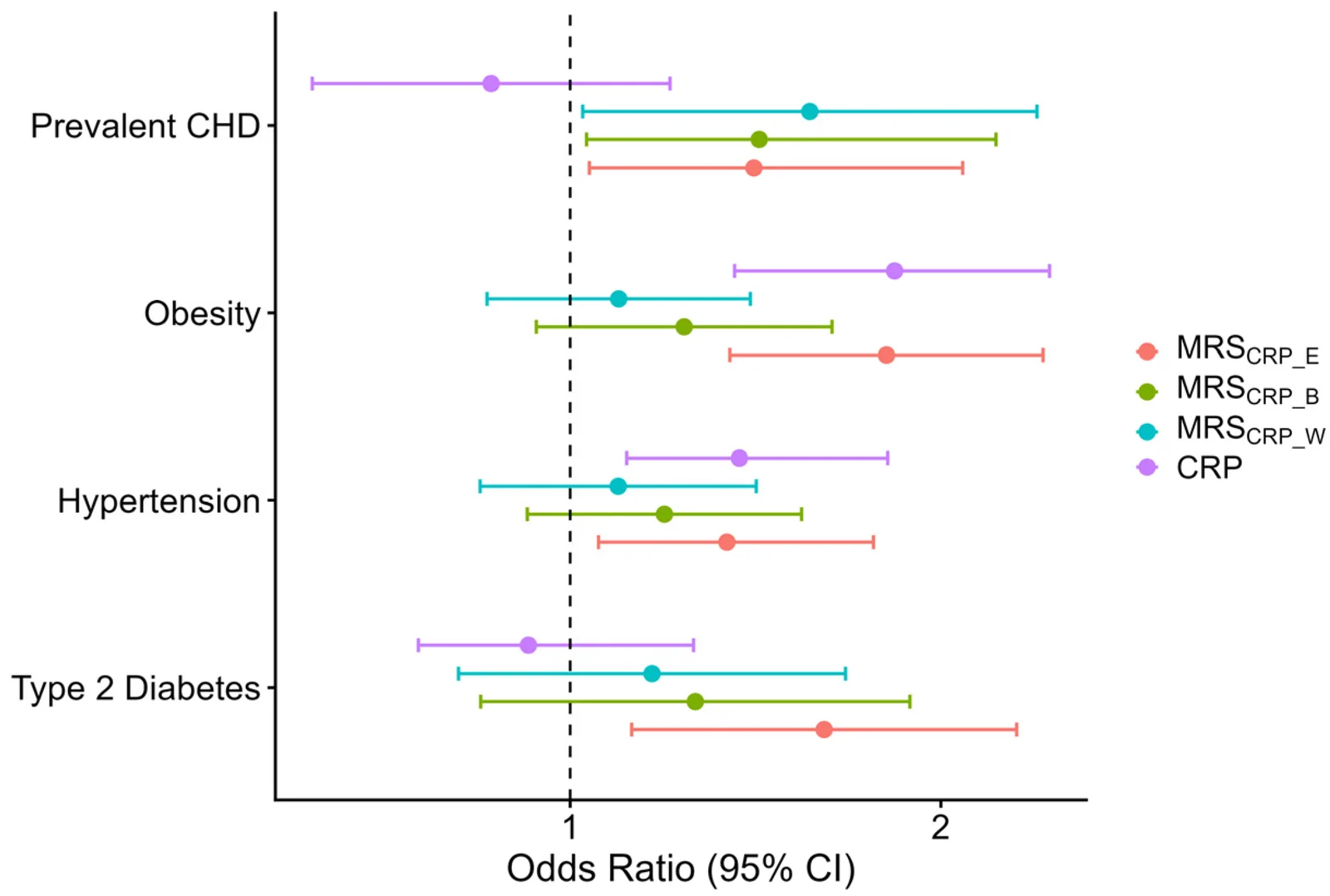
CRP measures associated with clinical phenotypes in the Emory Twin Study. Odds ratios with 95% confidence intervals based on the GEE model. The vertical dashed line indicates null values.

The associations of plasma CRP and MRS_CRP_ with binary cardiometabolic variables, including prevalent CAD, obesity, hypertension, and type 2 diabetes, are shown in Figure 3. In the generalized estimating equation (GEE) models testing associations between CRP, MRS_CRP_, and binary clinical phenotypes, only MRS_CRP_E_ was significantly associated with all tested traits. Plasma CRP was significantly associated only with obesity and hypertension. MRS_CRP_W_ and MRS_CRP_B_ exhibited similar effect sizes for prevalent CAD compared with MRS_CRP_E_ but showed weaker associations for all other clinical phenotypes. Linear mixed-effects models for continuous variables showed that CRP and MRS_CRP_E_ had comparable association strengths with the tested continuous phenotypes, whereas the other MRS_CRP_ had weaker associations. These results are available in Figure S1. 

**Figure 4:**
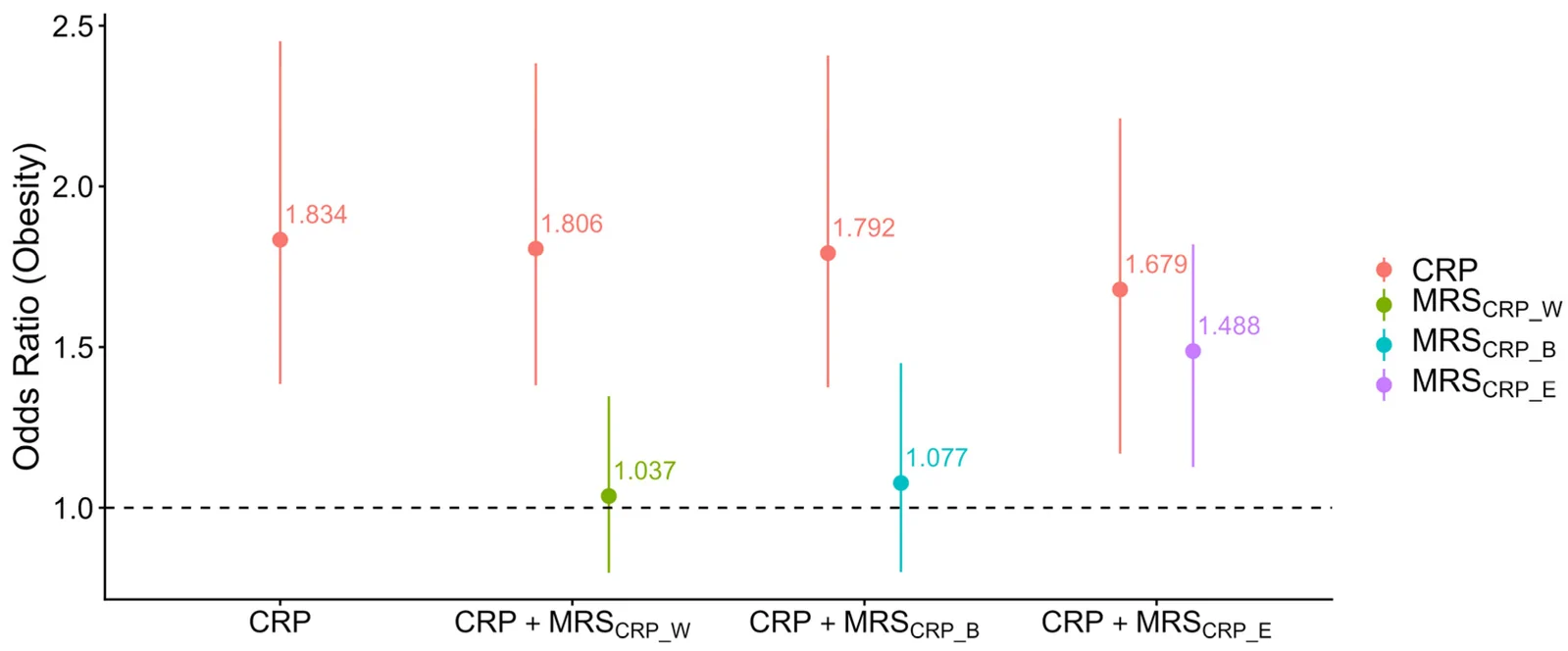
Association of CRP with obesity in models including MRS_CRP_, adjusting for age and co-twin status. Each CRP measure is labeled in a different color. Bars represent effect estimates and 95% confidence intervals. The horizontal dashed line indicates null values.

We further examined associations of MRS_CRP_ with clinical phenotypes, adjusted for plasma CRP to evaluate independent associations. The only MRS_CRP_ association that remained significant after adjusting for plasma CRP was the association of MRS_CRP_E_ with obesity (Figure 4, Figure S2). In the last model, where plasma CRP and MRS_CRP_E_ were included with adjustment for age and co-twins, significant positive associations were observed for both CRP and MRS_CRP_E,_ but not for other MRS_CRP_. In this model, plasma CRP and MRS_CRP_E_ were both significantly associated with obesity, with one-unit increases corresponding to 1.68 and 1.49 times the odds of obesity, respectively.

In conclusion, because of its consistent correlations with CRP and other inflammatory biomarkers and its significant and independent association with obesity, we decided to proceed with MRS_CRP_E_ as an indicator of low-grade inflammation for exploring longitudinal changes in PWH before and after ART.

### 2.2. MRS_CRP_ Longitudinal Changes in ADReSS

We evaluated the longitudinal changes in MRS_CRP_E_ in ADReSS. Demographic information for the ADReSS study populations at baseline and follow-up is summarized in Table 2. At both time points, 60.7% of participants were female. With an average of 6.5 years of follow-up, the mean age of participants increased from 34.6 years to 41 years. Mean CD4 counts increased from 259 cells/μL at baseline to 504 cells/μL at follow-up. Over 98% of participants in the study population received the fixed-dose combination tablet of efavirenz/emtricitabine/tenofovir disoproxil fumarate (EFV/FTC/TDF), while participants with contraindications to this regimen received zidovudine or abacavir in place of TDF and nevirapine in place of EFV. Because nearly all participants received the same first-line ART regimen, meaningful comparisons across treatment regimens were not feasible.

**Figure 5:**
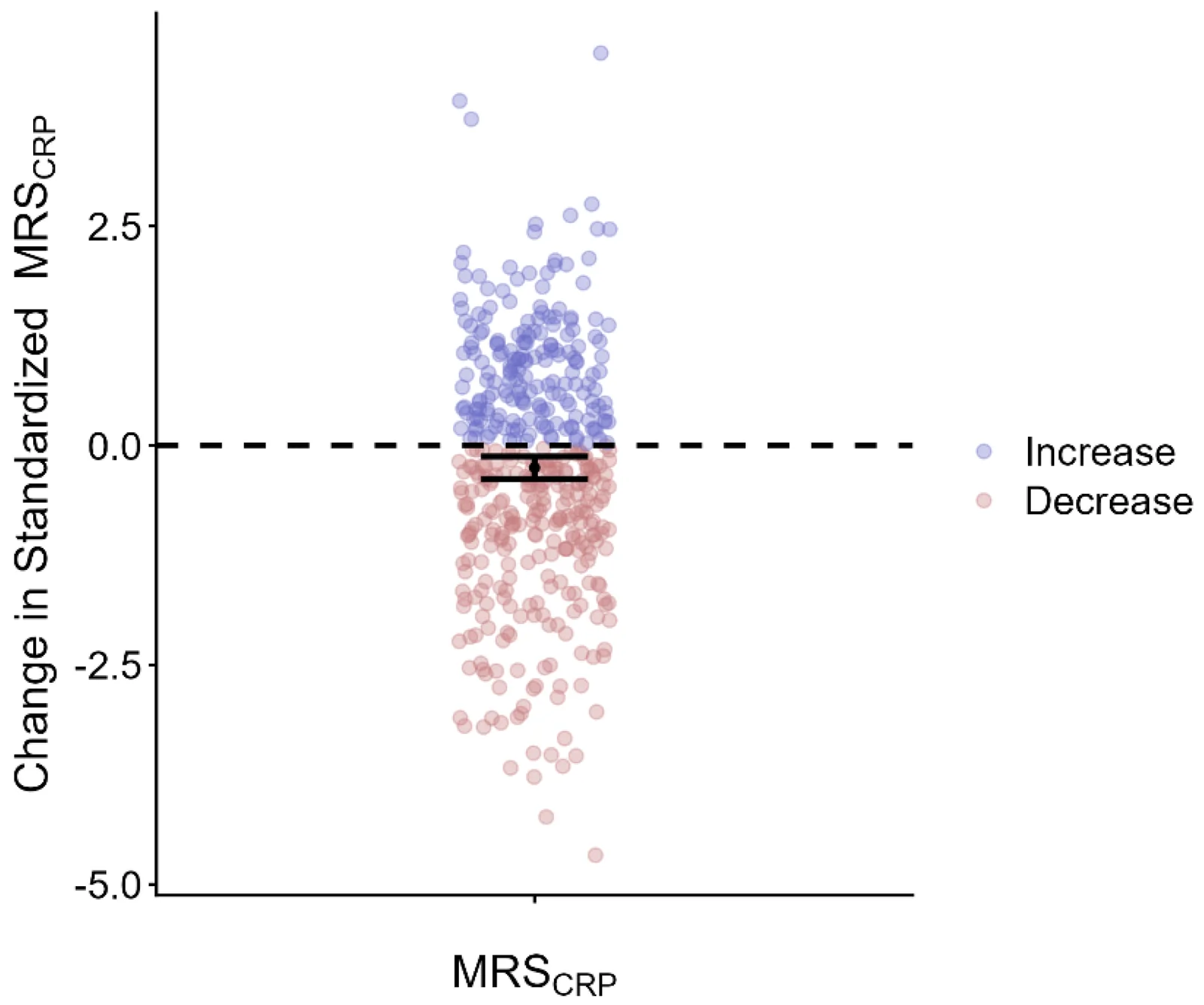
Changes in MRS_CRP_ after ART initiation among people with HIV. The y-axis represents the change in standardized MRS_CRP_. People with decreased MRS_CRP_ after ART compared with baseline are colored in red, and people with increased MRS_CRP_ after ART compared with baseline are colored in blue. The black dot represents the overall change in standardized MRS_CRP_ after ART, calculated as the mean difference between ART follow-up and baseline MRS_CRP_ values (paired *t*-test), and the error bar represents the corresponding 95% confidence interval. MRS_CRP_ values were jointly standardized. The reported change represents the difference in standard deviation (SD) units (mean difference: 0.254 SD units, paired *t*-test *p*-value: 5.47 × 10^−5^).

On average, MRS_CRP_E_ declined by 0.254 standardized units after ART, corresponding to a decrease of 0.254 standard deviations relative to the overall ADReSS cohort distribution (paired *t*-test, *p* = 5.47 × 10^−5^) (Figure 5). The average MRS_CRP_E_ predicted by a linear mixed-effects model with a random intercept for each participant showed an overall longitudinal decline in MRS_CRP_E_ (Figure S3). Predicted plasma CRP levels (mg/L) are presented in Figure S3. In sensitivity analyses, the overall decrease in MRS_CRP_E_ remained significant when the same linear mixed-effects model was adjusted for time-varying CD4 counts and their interaction with follow-up duration (Table S1). The results were still consistent after further adjustment for time-varying white blood cell proportions and methylation-based smoking status (Table S1).

## 3. Discussion

Our study evaluated MRS_CRP_, an epigenetic proxy of plasma CRP, as a potential marker for chronic inflammatory changes in PWH. We first evaluated three previously published MRS_CRP_ [23,24] in the Emory Twin Study for correlations with plasma CRP and other common biomarkers of inflammation. We further examined associations of MRS_CRP_ with selected cardiometabolic traits. We found that the MRS_CRP_ published by *Hillary* et al. using an elastic net regression model (MRS_CRP_E_) [24] performed best. MRS_CRP_E_ remained significantly associated with obesity after adjusting for plasma CRP, suggesting that it captured additional biological information. We proceeded to evaluate longitudinal changes in MRS_CRP_E_ among PWH in ADReSS, before and an average of 6.5 years after beginning ART. We found that, on average, MRS_CRP_E_ declined, although some increases were observed.

CRP is an important marker of chronic, systemic low-grade inflammation [29], but it also increases rapidly during acute infection or injury [30]. In comparison, MRS_CRP_, an epigenetic proxy of CRP, exhibits greater temporal stability [28]. In previously reported results, MRS_CRP_E_ showed a moderate correlation with plasma CRP, ranging from 0.27 to 0.53 across cohorts. In our study, MRS_CRP_E_ was consistently correlated with plasma CRP, as well as with IL-6, ICAM-1, and fibrinogen, outperforming the other MRS_CRP_ we evaluated.

The moderate correlation between MRS_CRP_E_ and plasma CRP in our study (Pearson correlation coefficient = 0.38) is expected because these measures may capture overlapping but different dimensions of inflammatory biology. Given that DNA methylation patterns can capture cumulative biological responses to environmental and disease-related exposures [31], while plasma CRP reflects circulating inflammatory status at a specific time point and can be influenced by transient inflammatory events [16,17,18,19,20], MRS_CRP_E_ captures a component of CRP-related inflammatory biology while also reflecting additional biological information, which may not be fully represented by a single CRP measurement. MRS_CRP_ should therefore not be interpreted as a replacement or direct surrogate for plasma CRP, but rather as a complementary epigenetic biomarker that provides additional information on chronic inflammatory processes. These results support the validity of MRS_CRP_E_ as an epigenetic proxy of systemic inflammatory status. These findings suggest that MRS_CRP_E_ captures not only biological information complementary to plasma CRP but also a broader systemic inflammatory burden.

MRS_CRP_E_ showed significant associations with cardiometabolic traits, including continuous measures (BMI, triglycerides, and HDL) and binary phenotypes (obesity, hypertension, type 2 diabetes, and prevalent CAD). Plasma CRP demonstrated similar directionality of associations with these traits, whereas other MRS_CRP_ showed weaker or attenuated relationships. Notably, the association between MRS_CRP_E_ and obesity remained significant after adjustment for plasma CRP. Together, these findings suggest that MRS_CRP_E_ captures components of systemic inflammatory burden not fully reflected by circulating CRP and may serve as an epigenetic marker of cardiometabolic-related inflammation.

As plasma CRP is prone to fluctuations with infection and other chronic diseases [16,17,32], MRS_CRP_ could have improved performance as a marker of long-term inflammation among people with a chronic infection. Previous studies have underscored how epigenetics can provide additional biological insight into inflammatory processes for PWH [21,22]. In our ADReSS cohort, the average longitudinal decrease in MRS_CRP_E_ was 0.254 standard deviation units, corresponding to an estimated reduction of 0.16 mg/L in plasma CRP during an average follow-up of 6.5 years after ART initiation. The lack of measured CRP limits our ability to directly reference changes in inflammatory status. However, CRP may be influenced by acute inflammatory events and therefore may not fully capture long-term inflammatory burden. A prior study reported a minimal median within-person change in plasma CRP levels after ART initiation (approximately a 1% reduction from pre-ART levels), compared with larger median reductions observed for other inflammatory biomarkers (ranging from 5% to 25%) [33]. Given that a prior EWAS has shown that ART initiation in PWH is associated with DNA methylation changes [34], the observed overall reduction in MRS_CRP_ in our study may capture longitudinal epigenetic changes associated with inflammatory regulation and provide additional insight into inflammatory changes during ART beyond what may be reflected by transient plasma CRP measurements. A previous epigenetic study has demonstrated that DNA methylation changes after ART are enriched in immune-related pathways [34], and other work has demonstrated the partial reversal of HIV-associated DNA methylation after ART [35]. While these studies have focused on immune-related processes, the longitudinal decline in MRS_CRP_ observed in our study, and the consistent decline after accounting for changes in CD4 count, white blood cell proportions, and methylation-based smoking status, are consistent with the possibility that ART-associated epigenetic changes may reflect improvements in inflammatory regulation. This may represent a complementary dimension of ART-induced epigenetic dynamics and could direct future epigenetic studies on inflammation for PWH under ART.

Our finding of longitudinal changes in MRS_CRP_ after ART among PWH aligns with emerging work suggesting that DNA methylation-based inflammatory signatures can capture cumulative inflammatory exposure [36,37]. Therefore, among PWH receiving ART, MRS_CRP_ may reflect longer-term inflammatory recovery and immune activation better than the transient fluctuations seen in circulating CRP levels. Overall, our results support the potential utility of methylation-based biomarkers as dynamic indicators of inflammation in PWH.

Our study has several limitations. First, due to a lack of relevant data, our analysis did not consider factors other than ART that could have effects on inflammation or epigenetic profiles. These could include other infections or unmeasured environmental or behavioral factors. Furthermore, the lack of a non-HIV longitudinal control group limits our ability to determine whether the observed changes in MRS_CRP_ were attributable to ART or reflected other longitudinal factors, such as aging, lifestyle changes, long-term medication use, and comorbidities. Although we performed sensitivity analyses adjusting for time-varying CD4 counts, estimated white blood cell proportions, and methylation-based smoking status, the observed decline in MRS_CRP_ may not fully capture the true changes in inflammatory status among PWH. In particular, the lack of measured plasma CRP levels in ADReSS can introduce uncertainty because changes in MRS_CRP_ may not directly correspond to changes in circulating CRP levels, limiting our ability to determine how closely longitudinal changes in MRS_CRP_ reflect changes in conventional inflammatory biomarkers. We observed some individuals with increased MRS_CRP_ after ART initiation, despite the overall decline observed in the cohort. This unexpected pattern may reflect residual confounding factors affecting DNA methylation levels or individual variability in inflammatory responses. Future studies incorporating more comprehensive phenotypic data will be needed to further investigate the factors contributing to longitudinal changes in MRS_CRP_. Our longitudinal analysis was conducted in a South African cohort. Therefore, the results may not be generalizable to other ancestry groups. Additionally, although MRS_CRP_ did not demonstrate sex- or ancestry-specific differences [23,24], further validation is needed to evaluate its cross-ancestry performance and its ability to capture longitudinal changes across diverse populations. Finally, the causal relationships between MRS_CRP_ and clinical traits require further investigation, especially in a longitudinal setting. 

## 4. Materials and Methods

### 4.1. Study Design

We selected three MRS_CRP_ from previous EWAS across multiple ancestry groups [23,24]. In the Emory Twin Study, we first evaluated the performance of each MRS_CRP_ to identify the optimal score. We calculated three MRS_CRP_ and assessed their correlations with plasma CRP and other inflammatory markers, and their associations with various clinical phenotypes known to be associated with inflammation. We further evaluated whether these associations persisted after adjustment for plasma CRP to assess whether MRS_CRP_ captured complementary information.

After selecting the best-performing MRS_CRP_, we applied this score to an independent cohort, the HIV/AIDS Drug Resistance Surveillance Study (ADReSS), to evaluate longitudinal changes before and after ART initiation among PWH. The study design workflow is presented in Figure 1.

### 4.2. The Emory Twin Study

The Emory Twin Study is a subset of the Vietnam Era Twin (VET) Registry, one of the largest national registries of male monozygotic (MZ) and dizygotic (DZ) twin pairs. Participants were enlisted by the US military during the Vietnam conflict and were mostly White [38]. Participants underwent paired, in-person baseline evaluations and blood collection at the Emory University General Clinical Research Center between 2002 and 2010. Follow-up evaluations took place on average 11.5 years later, either on-site or by phone [39]. Medical history and physical examinations were conducted for all participants at the in-person visits. All twin pairs were examined on the same day for consistency. In total, 352 twins (226 MZ and 126 DZ) with complete phenotypes and DNA methylation data were included in the present analysis to evaluate MRS_CRP_. The Emory Twin Study was approved by the Institutional Review Board at Emory University, and written informed consent was obtained from all participants.

Whole blood was collected and returned to the VET Registry overnight, where DNA was then extracted and stored for future research [40,41]. Plasma levels of inflammatory biomarkers, including high-sensitivity CRP (hs-CRP), interleukin-6 (IL-6), fibrinogen, and intercellular adhesion molecule-1 (ICAM-1), were quantified using commercially available ELISA kits (R&D Systems, Minneapolis, MN, USA). Enzymatic assays were used to quantify total cholesterol and triglyceride concentrations (Beckman Coulter Diagnostics, Fullerton, CA, USA). Body mass index (BMI) was calculated from each participant’s height and weight, measured in meters and kilograms. Obesity was defined as a BMI ≥ 30 kg/m^2^. Type 2 diabetes was defined by either the use of antidiabetic medications or a fasting glucose level > 126 mg/dL. A systolic/diastolic blood pressure measurement ≥ 130/80 mmHg was used to define hypertension. Prevalent coronary artery disease (CAD) was assessed through self-reported history of physician-diagnosed heart conditions or prior cardiovascular surgeries, including a history of myocardial infarction (MI), angioplasty, or coronary artery bypass graft (CABG).

Raw DNA methylation data were generated using the Illumina Infinium MethylationEPIC (850K) BeadChip (Illumina, Inc., San Diego, CA, USA) [42]. The preprocessing steps were performed as previously described [43]. Two probe designs were implemented: Infinium I probes measured methylated and unmethylated signals separately, whereas Infinium II probes used a single bead type to infer methylation status. Raw data were obtained from IDAT files and further processed using the *minfi* package in R [44]. Methylated and unmethylated signal intensities were extracted, and DNA methylation levels were quantified as beta values ranging from 0 (hypomethylation) to 1 (hypermethylation). Probes with a detection *p*-value greater than 0.01 were set to missing. Probes with more than 5% missing sites were removed. Samples with more than 10% missing methylation data (n = 4) were excluded. Quantile normalization was performed to correct batch effects. After these quality control procedures, 846,459 CpG sites quantified by beta values remained.

### 4.3. The KwaZulu-Natal HIV AIDS Drug Resistance Surveillance Study

The KwaZulu-Natal (KZN) HIV/AIDS Drug Resistance Surveillance Study (ADReSS) is a longitudinal cohort study of ART effects among ART-naïve PWH conducted in the province of KZN, South Africa. Recruitment took place at RK Khan Hospital and Bethesda Hospital in the province between 2014 and 2016 [45]. One thousand participants were enrolled in ADReSS, with an average of 6.5 years of follow-up. Among 616 samples with DNA methylation data, 440 had both baseline and follow-up measurements and were included in this analysis. Institutional Review Board approval was obtained from the University of KwaZulu-Natal, Emory University, and Mass General Brigham prior to the start of this study. All participants provided written informed consent.

Demographic and clinical data, including age, sex, and CD4+ T-cell counts, were collected at each study visit. All participants initiated ART after enrollment and received standard clinical care during the follow-up period. Detailed information on ART use, including medication type, duration of treatment, and the date of ART initiation, was also documented [45].

Blood samples were collected and processed for genome-wide DNA methylation profiling using the Illumina Human Methylation EPIC v2.0 (935K) BeadChip array (Illumina, Inc., San Diego, CA, USA) at each visit. This array type has greater robustness and adaptability than previous EPIC versions [46,47]. It includes over 900,000 CpG sites and provides more signals from 200,000 newly added probes at open chromatin and enhancer regions [48]. Baseline and follow-up samples were assayed during the same laboratory period; however, paired samples were not processed on the same array, plate, or batch. Samples were randomized by time point, sex, clinical site, and other relevant characteristics to minimize potential batch effects. DNA methylation preprocessing followed the pipeline described previously, with modifications for the EPIC v2.0 platform [49]. Technical variations, including dye bias, probe-type bias, slide, array, row, column, plate, and bisulfite-conversion batch effects, were addressed during preprocessing. Quantile normalization was performed separately within each of the six probe categories, and the positive controls included on each plate were evaluated. Quality control procedures were implemented to remove probes with a detection *p*-value greater than 0.01 and a missing rate exceeding 5%, ensuring an overall call rate above 90% for all samples. Beta values were calculated for the remaining 929,871 CpG sites that passed the preprocessing steps and quality control for further longitudinal testing of MRS_CRP_.

### 4.4. MRS_CRP_ Calculation

We selected three MRS_CRP_ that included published weights for different lists of CpG sites that had been associated with plasma CRP in previous multi-ancestry, epigenome-wide studies [23,24]. The first weights were obtained from an EWAS by *Wielscher* et al., who analyzed European, African, and South Asian cohorts using both Illumina450K and EPIC platforms and identified 1511 independent genome-wide significant CpG sites associated with CRP (MRS_CRP_W_) [23]. The other two weighted matrices were constructed from *Hillary* et al.’s EWAS, conducted in multi-ethnic cohorts across a broad age range using both Illumina 450K and EPIC arrays. Advanced algorithms were implemented to establish (1) a Bayesian penalized regression-based MRS_CRP_ (MRS_CRP_B_) and (2) an elastic net regression-based MRS_CRP_ (MRS_CRP_E_) [24].

Using the published weights from these three MRS_CRP_, we computed individual scores separately for each model. Individual DNA methylation beta values at each CpG site were multiplied by their corresponding weights and summed across all the required CpG sites for that MRS_CRP_. Missing CpG sites within individual samples were imputed using mean imputation based on the observed beta values of the corresponding CpG sites. CpG sites that were completely unavailable across all samples were primarily missing due to differences between methylation array versions [50], as some CpG sites included in the original MRS_CRP_ models were not present on the EPIC v2 array. These missing CpGs were imputed using the *mLiftOver* function from the *sesame* package [51].

### 4.5. Statistical Model in MRS_CRP_ Evaluation

The three MRS_CRP_ were evaluated in the Emory Twin Study. All skewed biomarkers were transformed to natural log scales to achieve a normal distribution. Linear mixed-effects models for each pair of MRS_CRP_ and plasma CRP were fitted with a random intercept for twin pair, and Pearson correlation coefficients were calculated from the model-derived estimates.

To further assess the biological relevance of MRS_CRP_ as epigenetic proxies of CRP levels, we also evaluated correlations of MRS_CRP_ with selected CRP-responsive inflammatory biomarkers, including IL-6, ICAM-1, and fibrinogen.

Due to the established connection between inflammation and cardiometabolic diseases [52,53], we also investigated associations with these phenotypes. We used linear mixed-effects models with random effects for twin relatedness to investigate associations of plasma CRP or MRS_CRP_ with cardiometabolic-related continuous measurements, including BMI (kg/m^2^), high-density lipoprotein (HDL) cholesterol (mg/dL), and triglycerides (mg/dL). Generalized estimating equations (GEE) were used to assess associations between plasma CRP or MRS_CRP_ and binary clinical variables (obesity, hypertension, type 2 diabetes, and prevalent CAD), accounting for twin-pair relatedness. All statistical models were adjusted for age. Each MRS_CRP_, as well as plasma CRP, was z-score-standardized before analysis to ensure comparability of effect sizes across models.

CRP is a marker of acute inflammation, but MRS_CRP_ is a more stable measure reflecting chronic, low-grade inflammation. We tested for independent effects of plasma CRP and each MRS_CRP_ on clinical traits, which would suggest complementary effects.

We selected the best-performing MRS_CRP_ based on its correlation with CRP and other inflammatory biomarkers, as well as its associations with cardiometabolic traits. The best-performing MRS_CRP_ was further evaluated for longitudinal changes in PWH on ART.

### 4.6. Statistical Model for Longitudinal Changes in MRS_CRP_

The MRS_CRP_E_ derived from the elastic net regression model from *Hillary* et al.’s study was selected for testing longitudinal changes among PWH from the ADReSS cohort [24]. MRS_CRP_E_ was z-score standardized jointly across both baseline and follow-up samples. Paired *t*-tests and linear mixed-effects models with random intercepts for each participant were performed to characterize longitudinal changes in chronic, low-grade inflammation measured by MRS_CRP_E_ after ART, while accounting for participants’ age, sex, and follow-up duration. Estimated average MRS_CRP_ levels were calculated to illustrate cohort-level longitudinal changes. Due to the lack of measured plasma CRP levels in ADReSS, we predicted the CRP levels using coefficients from the relationship between plasma CRP and MRS_CRP_E_ in the Emory Twin Study.

In a sensitivity analysis, the same linear mixed-effects model was extended to adjust for time-varying CD4 count and its interaction with the follow-up period to evaluate whether changes in MRS_CRP_E_ after ART were independent of CD4 changes. Additionally, due to the lack of measured white blood cell proportions (including CD4+ T cells, CD8+ T cells, B cells, natural killer cells, monocytes, and granulocytes), these cell-type proportions were estimated from DNA methylation data using the Houseman method implemented in the *minfi* package [54,55]. We then repeated the sensitivity analysis by further adjusting for these estimated cell-type proportions to examine the robustness of MRS_CRP_E_ changes. Furthermore, we adjusted for methylation-based smoking status (EpiSmokEr2) to account for lifestyle factors that could also lead to changes in MRS_CRP_E_ [56].

## 5. Conclusions

In summary, our results suggest further investigation of the potential utility of MRS_CRP_ in caring for PWH receiving ART. Longitudinal changes in MRS_CRP_ may reflect epigenetic responses associated with inflammatory recovery. As a DNA methylation-based marker, MRS_CRP_ may complement traditional biomarkers by capturing epigenetic responses to treatment. Future studies in larger and more diverse populations, including cohorts with directly measured inflammatory biomarkers, are needed to further validate the performance of MRS_CRP_ and determine its potential role in evaluating treatment response and long-term health outcomes among PWH.

## Figures and Tables

**Table 1:** Emory Twin Study participant characteristics (N = 352).

Variables	Values
Age in years (SD) *	55.7 (3.2)
High-sensitivity CRP (mg/L) (IQR) **	1.5 (2.6)
Triglycerides (mg/dL) (IQR) **	161.0 (109)
High-density lipoprotein (HDL) cholesterol (mg/dL) (IQR) **	36.9 (13.0)
BMI (kg/m^2^) (SD) *	29.8 (4.9)
Current smoker (%) ^†^	95 (27.0)
Obesity (%) ^†^	158 (44.9)
Prevalent coronary artery disease (CAD) (%) ^†^	40 (11.4)
Type 2 diabetes (%) ^†^	45 (12.8)
Non-Hispanic White (%) ^†^	346 (98.3)

* Variables are presented as means and standard deviations (SDs). ** Variables are presented as medians and interquartile ranges (IQRs). ^†^ Variables are presented as counts and proportions.

**Table 2:** ADReSS cohort participant characteristics (N = 440).

Variables	Baseline	Follow-Up
Age in years (SD) *	34.6 (9.7)	41.0 (9.7)
Female (%) ^†^	267 (60.7)	267 (60.7)
CD4 count/μL (SD) *	259 (151)	504 (259)

* Variables are presented as means and SDs. ^†^ Variables are presented as counts and proportions.

## Data Availability

The data are available from the National Health Laboratory Service in South Africa, but restrictions apply to the availability of these data, which were used under license for the current study, and so are not publicly available. Data are, however, available from the authors upon reasonable request and with permission of the National Health Laboratory Service in South Africa.

## Supplementary Materials

The following supporting information can be downloaded at https://www.mdpi.com/article/10.3390/epigenomes10030054/s1. Figure S1: CRP measurements associated with continuous clinical markers in the Emory Twin Study; Figure S2: Association of plasma CRP with clinical variables adjusting for each MRS_CRP_, age, and co-twin status in the Emory Twin Study; Figure S3: Longitudinal changes in standardized MRS_CRP_E_ and predicted plasma CRP levels (mg/L); Table S1: summary statistics of sensitivity analysis.
