# Supplementary material for: Longitudinal Changes in Inflammation-Related Methylation Risk Score for C-Reactive Protein Among People with HIV
Source: Epigenomes. 2026 Aug 12;10(3):54. doi: 10.3390/epigenomes10030054 (PMC13510981; doi:10.3390/epigenomes10030054)
Supplement: Supplementary file 1 [file epigenomes-10-00054-s001.zip › SuppFigure_Proofread.pdf]

Supplementary Figure S1. CRP measurements associated with continuous clinical markers in the Emory Twin Study

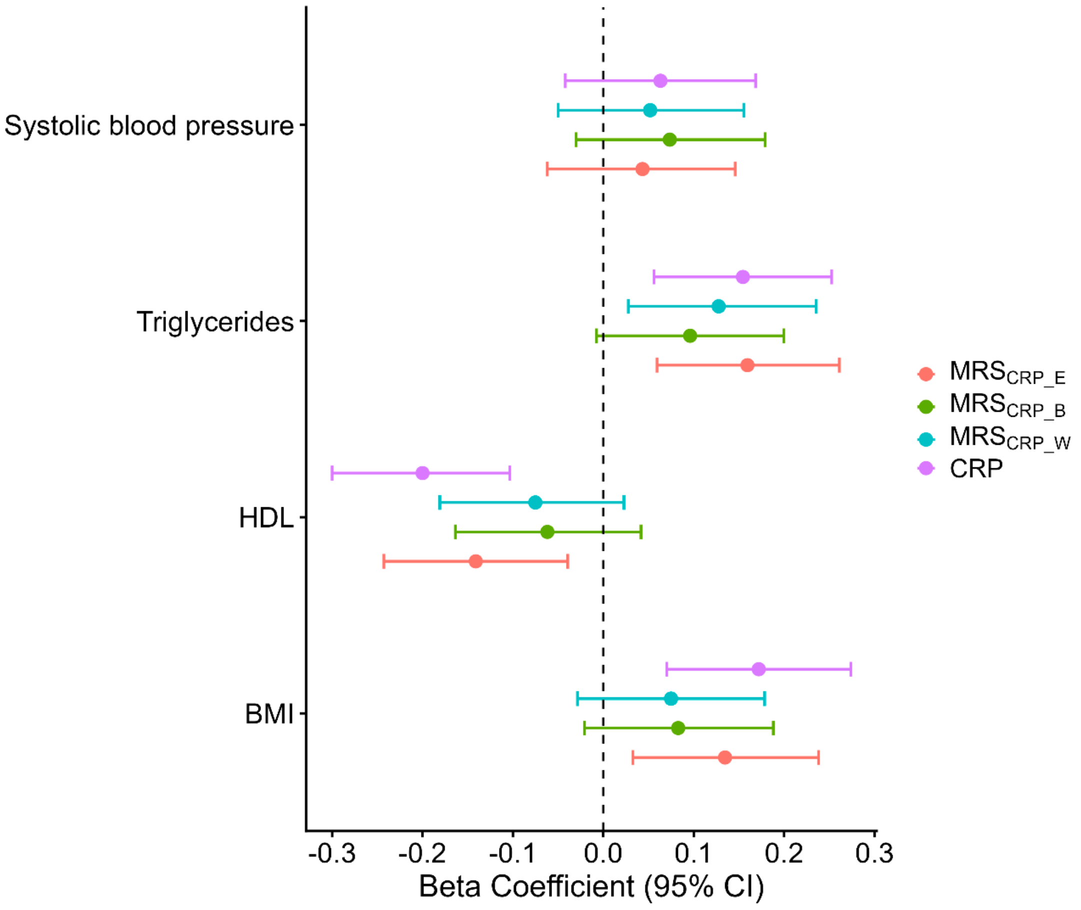

Beta coefficients with 95% confidence intervals based on linear mixed-effect models. The vertical dashed line indicates null values.

Supplementary Figure S2. Association of plasma CRP with clinical variables adjusting for each MRS_{CRP}, age and co-twin status in the Emory Twin Study.

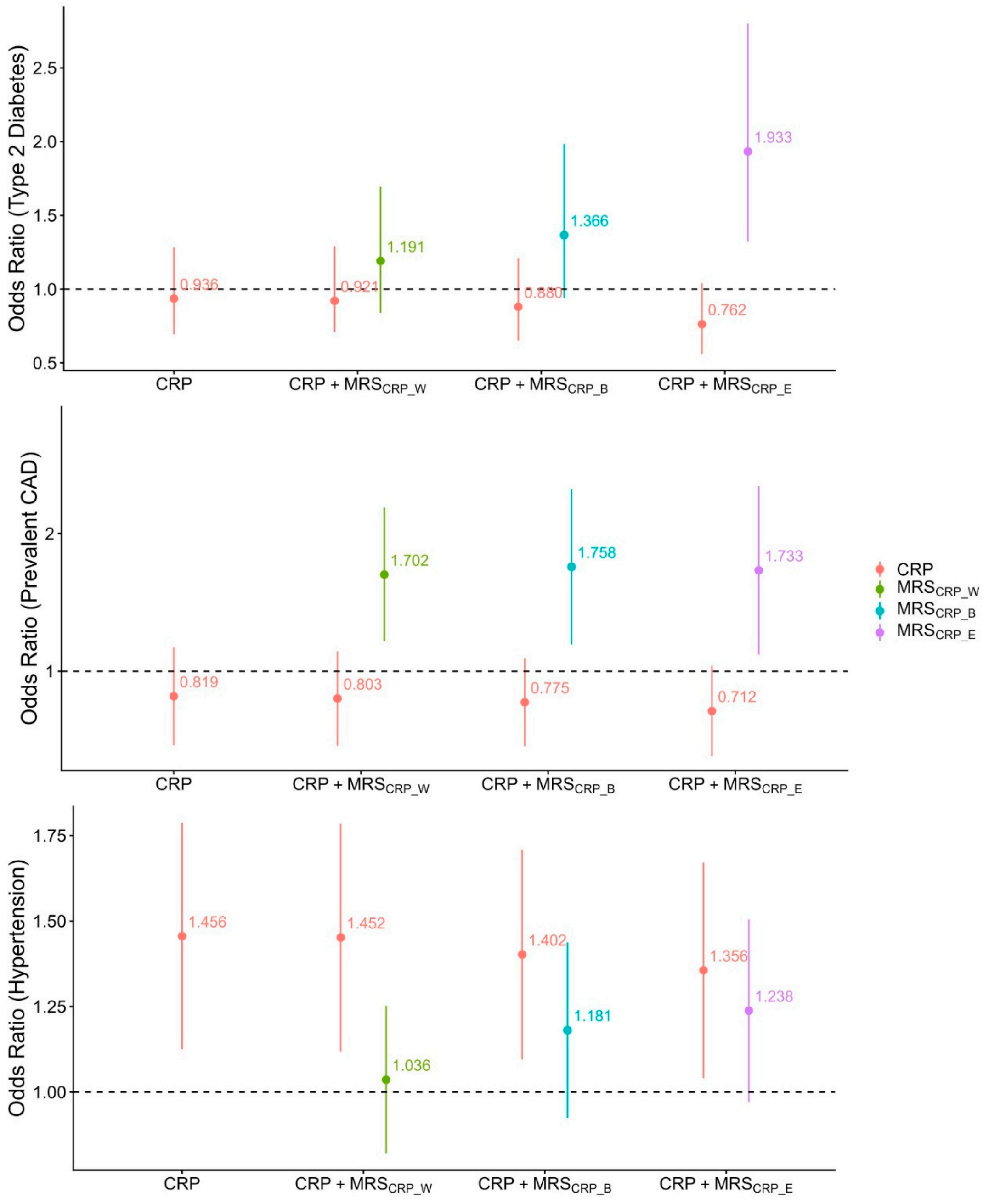

Odds ratios with 95% confidence intervals based on generalized estimating equation (GEE) model. The horizontal dashed line indicates null values.

Supplementary Figure S3. Longitudinal changes in standardized MRS_{CRP_E} and predicted plasma CRP level (mg/L).

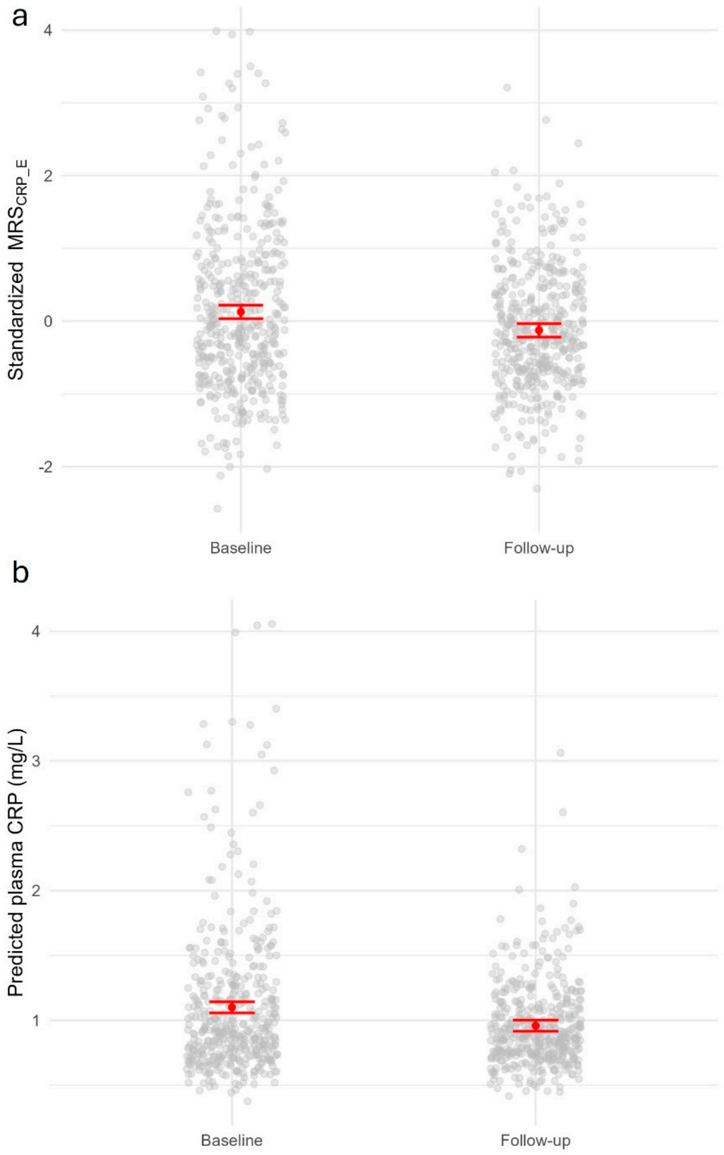

In panel a, the red dots and tails indicate the estimated average standardized MRS_{CRP_E} at baseline and follow-up after initiating antiretroviral therapy (ART) in HIV AIDS Drug Resistance Surveillance Study (ADReSS). The grey dots in the background are calculated standardized MRS_{CRP_E} for each individual. MRS_{CRP_E} were jointly standardized.

In panel b, the red dots and tails indicate the predicted average plasma CRP level (mg/L) at baseline and follow-up after initiating ART in ADReSS. The grey dots in the background are predicted plasma CRP level (mg/L) for each individual. The predicted plasma CRP level was estimated by linear mixed-effect model summary statistics from the Emory Twin Study.
