# Supplementary material for: Longitudinal Changes in Inflammation-Related Methylation Risk Score for C-Reactive Protein Among People with HIV
Source: Epigenomes. 2026 Aug 12;10(3):54. doi: 10.3390/epigenomes10030054 (PMC13510981; doi:10.3390/epigenomes10030054)
Supplement: Supplementary file 1 [file epigenomes-10-00054-s001.zip › SuppTable_Proofread.pdf]

	Model 1	Model 2	Model 3
Estimate (SE)	-0.21 (0.09)	-0.19 (0.08)	-0.30 (0.12)
p-value	0.02	0.03	0.01

Table S1: Summary statistics of sensitivity analysis

Estimate (SE): Beta coefficient representing the change in MRS_{CRP_E} at follow-up relative to baseline (reference), with standard error (SE), obtained from linear mixed-effects models.
Model 1: LME adjusting for time-varying CD4 count and its interaction with follow-up duration
Model 2: model 1 + time-varying white blood cell proportions (CD4+ T cells, CD8+ T cells, B cells, natural killer cells, monocytes, and granulocytes)
Model 3: model 2 + methylation-based smoking status
